# Determination of Controlled Self‐Assembly of a Paracrystalline Material by Homology Modelling with Hybrid NMR and TEM

**DOI:** 10.1002/chem.201701172

**Published:** 2017-06-26

**Authors:** Brijith Thomas, Jeroen Rombouts, Karthick Babu Sai Sankar Gupta, Romano V. A. Orru, Koop Lammertsma, Huub J. M. de Groot

**Affiliations:** ^1^ Leiden Institute of Chemistry Einsteinweg 55 2333CC Leiden The Netherlands; ^2^ Vrije University Amsterdam De Boelelaan 1083 1081 HV Amsterdam The Netherlands; ^3^ Department of Chemistry. University of Johannesburg Auckland Park Johannesburg 2006 South Africa

**Keywords:** mas nmr, phenazine, self-assembly, supra-molecular chemistry, tem

## Abstract

Controlling complexity, flexibility, and functionality of synthetic and biomimetic materials requires insight into how molecular functionalities can be exploited for steering their packing. A fused NDI‐salphen (NDI=naphthalene diimide) prototypic artificial photosynthesis material, DATZnS, is shown to be comprised of a phenazine motif, in which the alignment of electric dipole moments in a *P*2/*c* supramolecular scaffold can be modulated with bulky substituents. They can also be switched between parallel stacks of dipoles running antiparallel in the DATZnS‐H compared with parallel stacks of dipoles in polar layers running in opposite directions in the DATZnS(3′‐NMe) parent compound. Spatial correlations obtained from HETCOR spectra, collected with a long cross polarization contact time of 2 ms, reveal an antiparallel stacking for the DATZnS‐H homologue. These constraints and limited data from TEM are used to construct a structural model within the *P*2/*c* space group determined by the molecular *C*_2_ symmetry. By using homology modelling, a pseudo octahedral coordination of the Zn is shown to follow the packing‐induced chirality with enantiomeric pairs of the Λ and Δ forms alternating along antiparallel stacks. The model helps to understand how the steric hindrance modulates the self‐assembly in this novel class of fused materials by steric hindrance at the molecular level.

## Introduction

Spurred by the need for a zero‐emission‐energy system in the near future, scientists are working to mimic the primary processes of photosynthesis.^1^ Even though nature has given the blueprints for systems to convert energy from sunlight through photosynthesis, a principal challenge lies in finding suitable molecules that mimic the photochemical characteristics of natural photosynthesis for application in durable artificial systems by controlled self‐assembly.1g, 2 Recently, a structure‐based strategy was presented for the rational de novo design of biomimetic paracrystalline optical materials for application in molecular artificial photosynthesis electrodes. By applying TEM filtering in association with magic angle spinning (MAS) NMR, short‐ and medium‐range order was resolved and used to construct a high‐resolution packing model for paracrystalline fused naphthalene diimide (NDI)–salphen‐phenazine (DATZnS(3′‐NMe)), prepared by fusing NDI with Zn‐salphen, thereby generating a phenazine bridge.^3^ This material is a bioinspired prototypic artificial photosynthetic reaction center. It exhibits the properties of Zn‐salphen to form a metal–organic framework, whereas the NDI and the phenazine can be involved in enhanced π–π stacking. The wavelength at which photons are absorbed in the dyad can be adjusted with the functional groups so that it can cover the entire solar spectrum. Opto‐electronic tunability^4^ of the molecule makes this fused material a versatile building block for chemical engineering purposes.^5^ The aliphatic tails enhance the solubility of chemical precursors and allow for modification at a later stage, for example, by using them as a linker to attach self‐assembled antenna systems to functionalized electrode surfaces. The vacant orbitals present in the Zn^2+^ of the salphen can also accommodate an additional ligand. For instance, a catalyst can be attached through a coordination bond to the scaffold.^6^ Compact π–π stacking arising from the phenazine helps to form a rigid scaffold to attach such catalysts.^7^ The phenazine aromatic surface is flat and electron rich.^8^ This creates the possibility of attractive van der Waals and charge‐transfer interactions similar to supramolecular assemblies built from porphyrins.^9^ The properties and applications of these organometallic frameworks can be altered by changing the Zn^2+^ with other metals or by introducing a functionalized ligand. Considering that the fused salphen‐phenazine‐NDI motif has an electric dipole moment, this provides a handle for functionalization and chemical engineering of the electronic properties by modulated self‐assembly from steric control at the molecular level to steer the packing.

**Figure 1:**
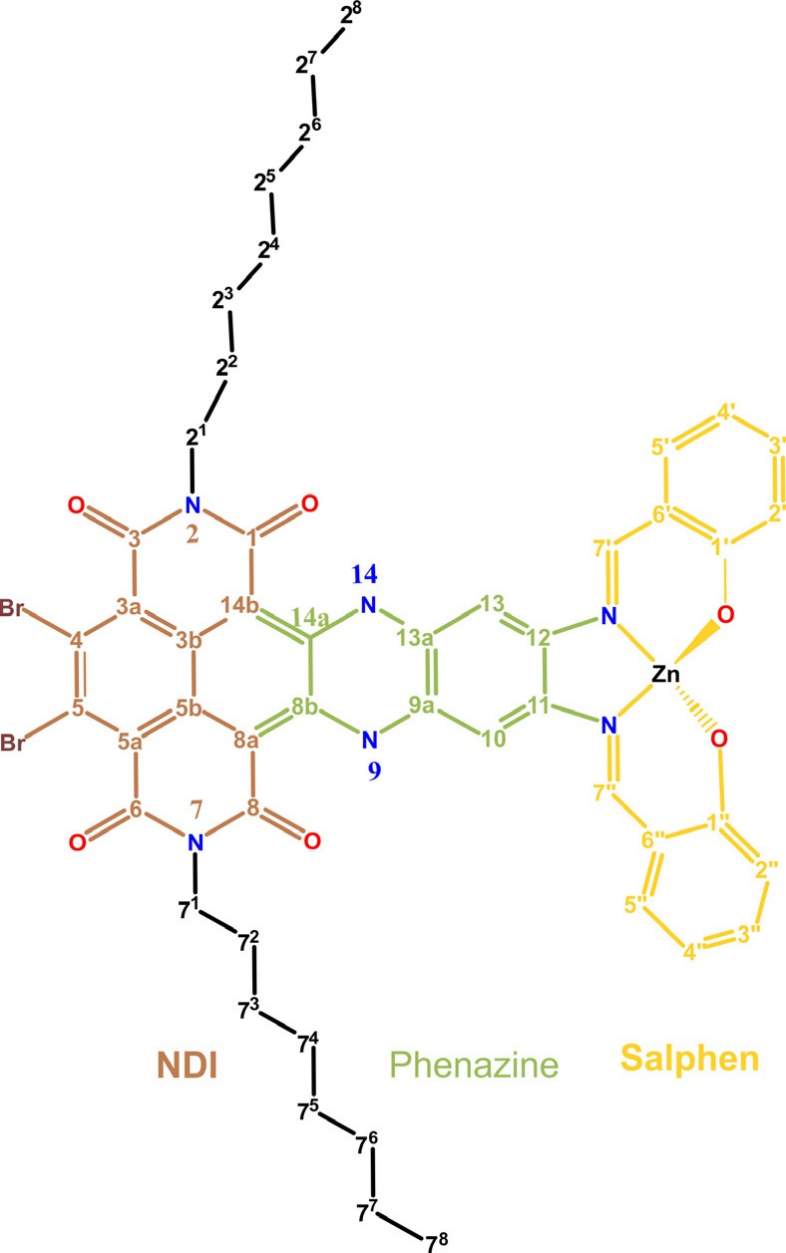
Chemical structure of the fused NDI‐zinc‐salphen‐based DATZnS‐H chromophore (salphen=bis‐salicylimide phenylene) molecule with numbering according to the IUPAC convention, which is followed throughout the manuscript. DATZnS chromophores are catching interest in the field of chemical design of light harvesting antenna molecules. These molecules are both robust and versatile with respect to optical tuning and other chromophoric chemical properties.

To mimic the engineering principles of nature as well as investigate and optimize the functional properties using theoretical and computational studies ahead of experimental realization, we have to understand how the dimensions and shapes of self‐assemblies are determined by steric and electronic‐packing factors.^10^ The DATZnS‐R model system with R=H, 3′‐NMe was set up de novo to mimic a chemically unrelated bacteriochlorophyll light‐harvesting system in natural chlorosomes and can be prepared with diverse functional groups to steer the packing. DATZnS(3′‐NMe) forms parallel stacks and antiparallel polar sheets by steric control to modulate the self‐assembly. Transfer of *C*_2_ molecular symmetry into packing symmetry was identified to be predominant in establishing a *P*2/*c* packing. Here we resolve a well‐determined molecular mechanism for controlling the formation of arrays of electric dipoles by determining a high‐resolution model of a chemical homologue, DATZnS‐H, which belongs to the same class of fused novel chromophore materials (Figure 1).^11^

Computational integration of MAS NMR spectroscopy with Cryo‐EM periodograms is opening a new horizon to high‐resolution visualization of supramolecular structures with well‐defined scaffolding and intrinsic heterogeneity at the molecular level.^10^, ^12^ By using TEM as a filter to determine reflection conditions, along with chemical shift information and distance constraints obtained by cross‐polarization MAS (CP/MAS) NMR ^1^H‐^13^C heteronuclear correlation spectroscopy data, short‐ and medium‐range order can be resolved to determine the space group and extrapolated to simulate a high‐resolution model for the supramolecular organization of a paracrystalline material. In this work, we extend the concept with a homology modeling step that allows to resolve how the paracrystalline packing adapts to structural variability. We determine the effect of the 3′‐NMe functionality at the molecular scale on the short‐range order. Intermolecular ^1^H‐^13^C distance constraints are obtained from frequency‐switched Lee–Goldburg (FSLG) heteronuclear correlation data collected with longer cross polarization contact times from the unlabeled material.^13^ The NMR constraints and DFT simulations point to *C*_2_ molecular symmetry of the DATZnS‐H, which can be accommodated with a racemic packing of the delta and lambda forms in the *P*2/*c* unit cell with the two‐fold crystal symmetry axis running along the *C*_2_ molecular symmetry axis that was determined for the parent compound. The packing and unit cell parameters were optimized using molecular mechanics, and the ^1^H‐^13^C constraints and a principal reflection in reciprocal space deduced from the electron microscopy were reproduced to validate the packing. The data provide converging evidence that steric control by the bulky 3′‐NMe substituent allows for a switch between parallel and antiparallel stacking of the DATZnS motif.

## Results

Moderately sized molecules like DATZnS‐R with low symmetry and few hydrogens on the extended network of conjugated aromatic rings, make it feasible to identify selective intermolecular polarization transfer events, to spatially probe the structure.^13^ For the DATZnS‐H, ^13^C 1D CP/MAS NMR resonances are well dispersed, over 170 ppm, due to different functionalities, because there are four carbonyl groups, two phenoxy rings, and two imide groups present in the fused NDI‐zinc‐salphen‐based chromophore (S1). Narrow NMR lines point to a well ordered microstructure.

**Figure 2:**
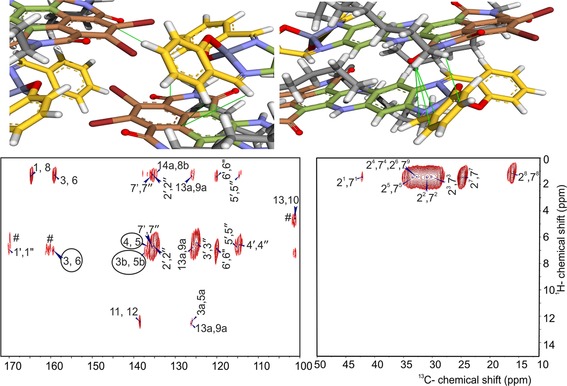
Contour plot sections of ^1^H‐^13^C heteronuclear dipolar correlation spectra collected with 2 ms cross polarization contact time, showing long range correlations signals. The data were recorded in a field of 17.6 T with a spinning frequency of 13 kHz. The spectrum is divided over two panels, aliphatic on the right and aromatic on the left. Folding of the tails along the phenazine can be deduced from a set of correlation peaks between the ^1^H from the aliphatic tail and the ^13^C on the phenazine backbone. Formation of a pseudo octahedral recognition motif involving the Zn^2+^ and the two bromine of adjacent molecules transpires from an intermolecular correlation indicated by circles arising from the interaction between salphen and NDI. # indicates the side bands.

The ^13^C chemical shifts σCsolid of the DATZnS‐H were tentatively assigned by analogy to the NMR response of DATZnS(3′‐NMe), and the assignment was validated with computational chemical shifts, which are well in line with the data (Table 1).1g Assignments of the primary, secondary and tertiary carbon atoms can be confirmed with the aid of ^1^H‐^13^C heteronuclear dipolar correlation data acquired with a short cross polarization contact time of 0.256 ms, to limit long range intermolecular transfer (*S*2). For the ^13^C, the aliphatic response is shown between 10 ppm and 50 ppm, whereas the aromatic region covers the range from 100 to 170 ppm. The aromatic CH protons are partially resolved in the ^1^H‐^13^C spectra (Figure 2). The terminal methyl group on the alkyl chain has a characteristic shift of 13.7 ppm, whereas the alkyl group attached to the electronegative nitrogen resonates with a chemical shift of 40.7 ppm. The resolved signal around 22.6 ppm is attributed to the 2^7^, 7^7^ responses that are essentially indistinguishable, confirming a translation of *C*_2_ symmetry into packing symmetry with a twofold axis. The 13, 10 in the phenazine ring are also symmetry related and have characteristic ^13^C chemical shifts of 100.1 ppm.

**Table 1:** Experimental MAS solid state shifts compared with computational results from Gaussian 03 calculations for monomeric DATZnS‐H.

Position	σCsolid	σCcalc
1, 8	163.6	162.5
3, 6	158.3	157.8
4, 5	135.4	152.3
14b, 8a	99.0	101.7
14a,8b	133.5	136.7
3a, 5a	124.8	125.1
3b, 5b	136.1	130.7
13a, 9a	125.0	128.1
13, 10	100.3^[a]^	99.1
11, 12	137.5	140.4
1′, 1′′	169.1	176.2
2′, 2′′	133.9^[a]^	129.2
3′, 3′′	123.6^[a]^	128.4
4′, 4′′	113.6^[a]^	104.8
5′, 5′′	113.9^[a]^	120.6
6′, 6′′	118.9	107.8
7′, 7′′	134.3^[a]^	139.5

[a] Assignment based on the computational chemical shifts and dipolar correlation data collected with a short mixing time of 0.256 ms.

In the ^1^H‐^13^C data collected with a cross polarization contact time of 2 ms, there are many long‐range correlation signals arising from heteronuclear dipolar transfer between proton and carbon nuclei, which can be intermolecular or between different parts of the DATZnS‐H molecule (Figure 2).^12^ The protons attached to the tertiary carbon nuclei are well‐resolved in the 2D data collected with a short cross polarization contact time of 256 μs (Figure S2). The difference is important for the selective detection of long‐range correlations at a cross polarization time of 2 ms, to resolve the spatial rearrangement of the DATZnS‐H molecules in the *P*2/*c* packing relative to the structure of the parent compound.

Long‐range intramolecular correlations are observed between protons attached to the carbon atoms on the tail and the 1, 8, 3, 6 carbonyl groups. For instance, the carbonyl group could get polarization transferred from the 2^1^, 7^1^ CH_2_ alkyl groups over intramolecular distances less than 4 Å, depending on the molecular conformation. Transfer of polarization from protons on the alkyl chain to 14a, 8b, 13a, 9a, 6′, 6′′, 5′, 5′′, 2′, 2′′, 7′ and 7′′ ^13^C gives information about the folding and positioning of the tails in the packing. Although transfer to 14a, 8b, 13a, 9a ^13^C could be intramolecular or intermolecular, the transfer to 6′, 6′′, 5′, 5′′, 2′, 7′ is intermolecular and points towards antiparallel stacking (Figure 2). Heteronuclear correlation signals involving the quaternary ^13^C in the highly unsaturated NDI part and the salphen are very useful for structure determination, because there are no protons on the NDI motif for intramolecular transfer. Extensive polarization transfer from aromatic protons on the salphen part to 4, 5, 3, 6, 3b, and 5b quaternary ^13^C nuclei corroborate an antiparallel stacking arrangement (Figure 2). For a cross polarization time of 2 ms, a spatial correlation is observed between the 14‐NH proton and 3a and 5a ^13^C. A rapid buildup of signal indicates it concerns primarily intramolecular transfer (data not shown).

**Figure 3:**
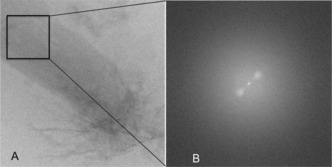
A) TEM image of the DATZnS‐H loaded on a carbon grid revealing a lamellar arrangement. B) Fourier transform of the selected region indicated with a black square shows a 1.83 nm periodic repetition.

Stripes observed in the TEM image of Figure 3 A lead to a Bijvoet pair of strong reflections at a distance of 1/1.83 nm in the Fourier transform of Figure 3 B. They occur in various images, and reveal periodic repetition of the molecule in a lamellar arrangement (Figure S3). Since no other distinct reflection could be observed in the TEM images, there is considerable heterogeneity in the sample and the packing.

## Discussion

**Figure 4:**
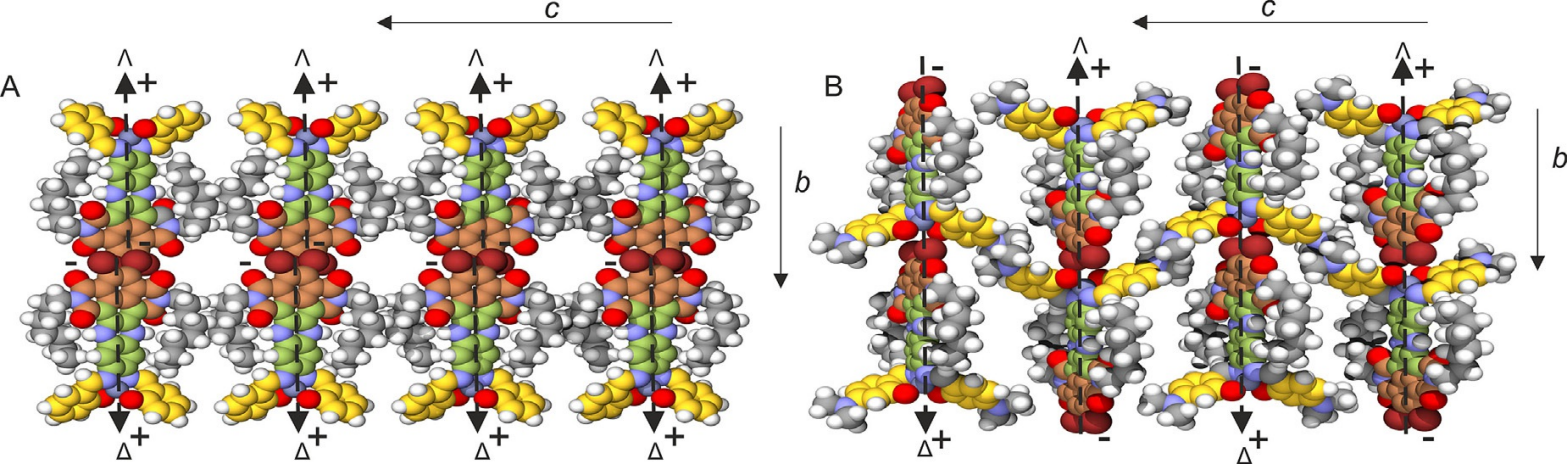
Proposed structure of antiparallel stacked DATZnS‐H (A) in a 3D packing with the space group *P*2/*c*. In comparison with the parent structure DATZnS(3′‐NMe) (B), the dipole moments are compensated between the stacks and antiparallel rows of head to tail in DATZnS‐H (A). Considering that the only difference between the two compounds are the NCH_3_ functional groups, chemical control over the dielectric characteristics at the supramolecular level is achieved.

Considering that the two halves of the molecule yield only one set of NMR signals, the data reveal molecular symmetry, either achiral σ_v_ or chiral *C*_2_, with the latter being the lowest in energy according to the DFT calculations. Hence, both NMR and modeling favor a two‐fold molecular axis, whereas the molecules should form lamellar packing according to the TEM. To arrive at a high‐resolution model for the packing, we follow a homology approach starting from the DATZnS(3′‐NMe) structure published previously.1g The DATZnS‐H was positioned in the cell on the two‐fold axis. Two settings were considered, the original setting with parallel stacking and a setting, in which the *a* and *c* axis were interchanged to obtain a structure with antiparallel stacking (Figure S4). Optimizations of unit cell parameters and molecule were performed for both settings and it was found that the antiparallel stacking is 37 kcal mol^−1^ lower in energy with parameters *a=*1.47 nm, *b=*1.83 nm, *c=*9.6 nm, *α*=90°, *β*=109° and *γ*=90° (Figure 4).

The extensive polarization transfer to 4, 5, 3, 6, 3b, and 5b from aromatic protons on the salphen part to the NDI part indicated in Figure 2 with a circle validates the antiparallel stacking, which has short distances of ≈3.5‐4 Å between the salphen protons and NDI carbons corresponding with the transfer range for heteronuclear correlation signals determined with quantitative simulations of the LGCP signal buildup curve.1g The alternative, recognition between the NDI and the salphen part with extended overlap in the parallel stack model is difficult to reconcile with the characteristic heteronuclear transfer in Figure 2. This contrasts with the parallel stacking deduced for the DATZnS(3′‐NMe) homologue, in which only correlations were observed between the NDI motif and the dimethylamine functionalities, which are proposed to play a decisive role in steering the packing configuration of the latter compound. When parallel stacking is imposed for the DATZnS‐H, not only the enthalpy is higher than for antiparallel stacking because of steric hindrance, but also the correlations detected in Figure 2 would require transfer over larger distances beyond 5 Å, which is unlikely. Finally, the heteronuclear correlation data for the DATZnS(3′‐NMe) homologue do not exhibit the characteristic correlations indicated with the circle in Figure 2.1g Thus, the heteronuclear transfer from ^1^H of the salphen to ^13^C of the NDI can be considered a decisive characteristic for structure determination to distinguish between parallel and antiparallel stacking.

The distance between two molecules in the direction perpendicular to the plane of the molecule is 4 Å,^14^ indicating strong π–π stacking. The tails are projecting outwards into the space between two stacks (Figure 4 B). The presence of the carbonyl groups and the nitrogen in the NDI with the lone pair induces an extended π‐electron delocalization.^15^ The electropositive Zn^2+^ in the Λ and Δ chiral salphen functionalities produce a cavity to which the electronegative bromine of the next molecule along the *b* axis is attracted to form the pseudo‐octahedral recognition motif, similar to the DATZnS(3′‐NMe) homologue (Figure 2 and 4 B). This electrostatic attraction can stabilize the packing along the axial direction of the molecule. The pseudo octahedral coordination of the Zn follows the packing induced chirality with enantiomeric pairs of the Λ and Δ forms alternating along the antiparallel stack.

In the TEM pattern of Figure 3, the stripes are from alternating regions of high electron density containing Zn^2+^ with less electron‐dense regions of phenazine. Along the antiparallel stack the dipole moments are aligned in opposite directions, which probably constitutes a less robust scaffold of the packing than for the DATZnS(3′‐NMe) parent compound. With the dipoles compensated at the level of the antiparallel arrangement within the stack, there is no emerging electrostatic component for stabilization of planes. This may explain why the DATZnS‐H structure appears much less ordered in the TEM, with only the two reflections of the long repeat along the *c* axis in the Fourier transform. Tight packing of dyads within the self‐assembled stacks provides a high chromophore density to harvest the solar light efficiently. The pseudo‐octahedral recognition motif is a characteristic of the supramolecular packing, and is consistent with the antiparallel arrangement that transpires from the spatial correlation peaks between salphen and NDI. The structure accommodates the folding of the tails along the phenazine, in line with the ^1^H‐^13^C dipolar correlation data collected with the longer cross polarization contact time of 2 ms. Steric hindrance from the salphen part restricts parallel stacking. Polymorph analysis shows that the π–π stacking motif remains intact, and it remains as the basic building block. The simulation of the TEM diffraction pattern from the high resolution model generates the strong pair from the lamellar arrangement as observed in the experiment with the distinct repetition of 1/1.8 nm (data not shown).^16^

## Conclusions

Based on the energy, density, intermolecular correlations from CP/MAS, reflection spots in the Fourier transform of the TEM image, and homology modelling, we converge upon an antiparallel stacking for DATZnS‐H forming lamellar sheets in a *P*2/*c* packing arrangement. Pseudo‐octahedral coordination of the Zn^2+^ and *C*_2_ molecular symmetry produces the twofold axis, whereas the two enantiomeric forms Λ and Δ produced by a *c*‐glide symmetry operation lead to a racemic packing with the alkyl chains folded along the phenazine. This structure is a homologue of the DATZnS(3′‐NMe) structure, and suggests that the NCH_3_ functional group can be used to steer the aggregation from a parallel sheet in DATZnS(3′‐NMe) to an antiparallel sheet in DATZnS‐H. Hence the packing of fused NDI‐salphen chromophores forming a phenazine motif with a dipole moment in a *P*2/*c* supramolecular scaffold can be steered by chemical substituents between antiparallel dipoles and parallel dipoles in a sheet. This concept paves the way for the chemical design of supramolecular scaffolds for light harvesting and charge separation, on the way to organic solar fuel cell device concepts that can be programmed to quench the internal field in an antiparallel arrangement, for example, for light harvesting, or exploit the internal field from aligned dipole moments, for example, for the injection of charge in catalysts for water splitting.

## Experimental Section

8,9‐Dibromo‐5,12‐dihydro‐5,12‐diazatetracene di‐*n*‐octylimide‐diamine intermediate (103 mg, 0.13 mmol), prepared from 2,3‐(*p*‐toluenesulfonamido)‐8,9‐dibromo‐5,12‐dihydro‐5,12‐diazatetracene di‐*n*‐octylimide was dissolved in dry, degassed DMF (9 mL) under an Ar atmosphere and heated to 80 °C in the dark.3a In a separate flask, salicylaldehyde (40 mg, 0.33 mmol) and zinc acetate dihydrate (265 mg, 1.32 mmol) were dissolved in dry, degassed DMF (5 mL) and kept under Ar. This mixture was stirred for 5 minutes and added to the hot DMF solution by means of a syringe. After 6 hours, the reaction mixture was cooled down to room temperature, diluted with 5 mL H_2_O and stored overnight at −20 °C to induce precipitation. The dark blue precipitate was collected on a filter and washed with water, ethanol, and dichloromethane to afford (after vacuum drying) 89 mg of a dark blue solid (59 % yield from 8,9‐dibromo‐5,12‐dihydro‐5,12‐diazatetracene di‐*n*‐octylimide‐diamine). M.p. >300 °C; IR (ATR FTIR): $\tilde{\nu }$ =2953, 2922, 2851, 1686, 1572, 1528, 1499, 1448, 1431, 1315, 1281, 1225, 1200, 1173, 1150, 1128, 1105, 1076, 1030, 964, 914, 847, 584 cm^−1^.

The solid‐state CP/MAS spectra were recorded with a Bruker AV‐750 MHz spectrometer, equipped with 4 mm triple resonance CP/MAS probes in dual ^1^H‐^13^C mode with a spinning frequency of 13 kHz±5 Hz. The data were collected at a sample temperature of 298 K. The magic angle was set using the ^79^Br resonance from KBr. The pulse sequence for the 2D heteronuclear polarization transfer experiment is shown in Figure S5. The 2D sequence starts with a magic angle preparation pulse on the ^1^H channel. After that protons are allowed to evolve for time *t_1_* with Phase Modulated Lee–Goldburg irradiation to suppress ^1^H homonuclear dipolar couplings. This is followed by transfer of the magnetization from the protons to carbons by a cross polarization step.^13^, ^17^ The two‐pulse phase modulation (TPPM) Scheme was used to decouple proton spins during acquisition while the ^13^C free induction decays (FIDs) were recorded in the *t_2_* domain.^18^ For short‐range polarization transfer from ^1^H directly bonded to ^13^C, a cross‐polarization contact time of 0.256 ms was used, and for longer range polarization transfer and the detection of intermolecular correlations, a cross‐polarization contact time of 2 ms was employed. The ^1^H chemical shift was calibrated with a ^1^H‐^13^C spectrum of solid tyrosine.HCl17b and a scale factor of 0.57 for the FSLG was verified. The data were processed using Bruker TopSpin 3.2 software (Bruker, Billerica, MA). 128 scans were collected for each of the 128 steps in the ^1^H dimension.

Computational chemical shifts were obtained with the Gaussian 03 software package (Gaussian, Inc., Wallingford, CT) using the Becke, Lee, Yang, and Parr (BLYP) exchange‐correlation functional with 6–311G basis set.^19^ The molecule was geometrically optimized prior to NMR chemical shift calculation.

Biovia Materials Studio Suite (Biovia, San Diego, CA) was used for computational modeling. A monomer structure of the DATZnS‐H core without the aliphatic tails was obtained by optimization with Gaussian 03 (Gaussian, Inc., Wallingford, CT) and placed in the *P*2/*c* unit cell determined for the homologue as described in the results section. Optimization of the cell parameters was performed with the FORCITE module. For geometry optimization the “smart” algorithm setting was used and a convergence tolerance of 0.001 kcal mol^−1^ for energy and 0.5 kcal mol^−1^ Å^−1^ for the force were applied. For the full molecule including the tails, DMol^3^ calculations were conducted to estimate the ESP charges. The generalized gradient approximation (GGA) with the Perdew‐Burke‐Ernzerhof (PBE) functional with double numerical atomic orbital augmented by a polarization *p*‐function (DNP) basis set was used for DMol^3^ calculations.

For electron microscopy, samples dissolved in ethanol were applied to a carbon grid at 83 K with a Vitrobot vitrification system (FEI, Hillsboro, OR). Electron microscopy was performed with a Tecnai G2 Polara electron microscope (FEI, Hillsboro, OR) equipped with a Gatan energy filter at 115 000 magnification (Gatan, Pleasanton, CA). Images were recorded in the zero‐loss imaging mode, by using a slit width of 20 eV, with a slow‐scan CCD camera at 1 micrometer under focus, to have optimal phase contrast transfer at 300 kV.

## Conflict of interest

The authors declare no conflict of interest.

## Supporting information

As a service to our authors and readers, this journal provides supporting information supplied by the authors. Such materials are peer reviewed and may be re‐organized for online delivery, but are not copy‐edited or typeset. Technical support issues arising from supporting information (other than missing files) should be addressed to the authors.

Supplementary
