# Supplementary material for: Determination of Controlled Self‐Assembly of a Paracrystalline Material by Homology Modelling with Hybrid NMR and TEM
Source: Chemistry. 2017 Jun 26;23(39):9346–51. doi: 10.1002/chem.201701172 (PMC5519927; doi:10.1002/chem.201701172)
Supplement: Supplementary file 1 — Supplementary [file CHEM-23-9346-s001.pdf]

CHEMISTRY
A European Journal

Supporting Information

Determination of Controlled Self-Assembly of a Paracrystalline Material by Homology Modelling with Hybrid NMR and TEM

Brijith Thomas,[a] Jeroen Rombouts,[b] Karthick Babu Sai Sankar Gupta,[a] Romano V. A. Orru,[b] Koop Lammertsma,[b, c] and Huub J. M. de Groot*[a]

chem_201701172_sm_miscellaneous_information.pdf

1 1D ^{13}C CP/MAS spectrum of DATZnS-H recorded at 750 MHz S2

2 HETCOR spectra of the DATZnS-H at a short mixing time of 0.256 ms S2

3 TEM images of the molecule in different orientation S3

4 Stacking of the molecules S4

5 Pulse sequence used for the HETCOR experiment S4

6 1D slices along the ^{1}H dimension S5

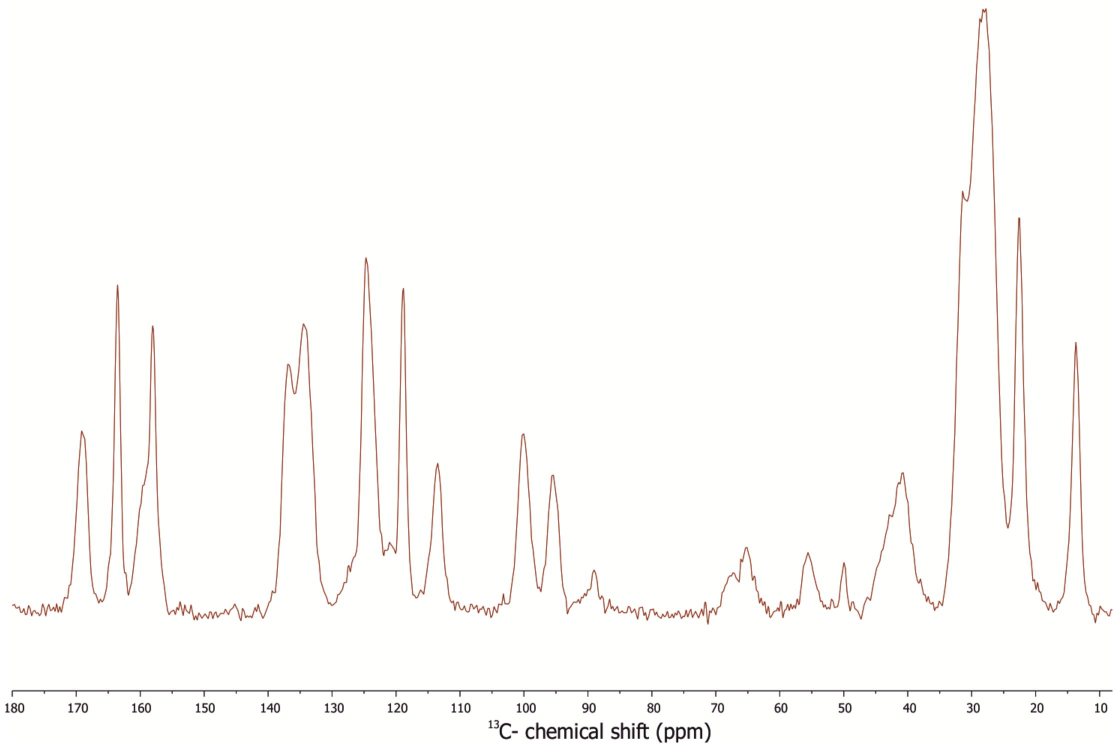

S1 1D ^{13}C CP/MAS spectrum of DATZnS-H recorded at 750 MHz.

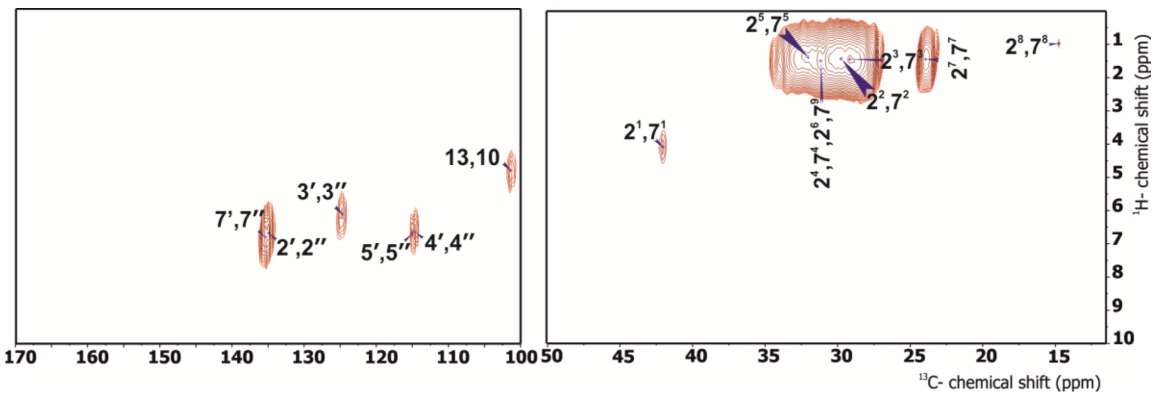

S2 HETCOR spectra of the DATZnS-H at a short mixing time of 0.256 ms.

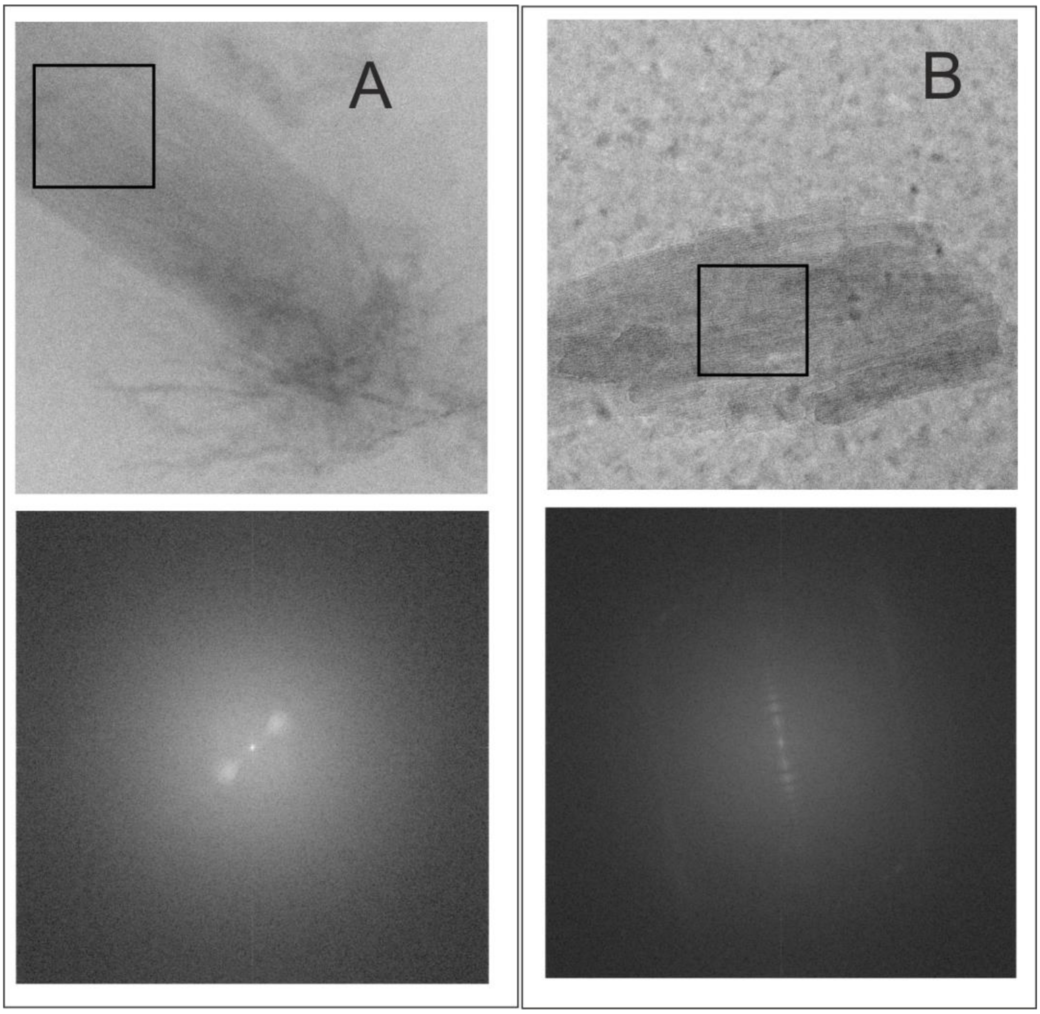

S3 TEM images of the molecule in two different orientations and their Fourier transform. Panel A points to a lamellar structure with a Bijvoet pair in the Fourier transformation of the TEM image, while in the panel B the evidence for polymorphism is obtained, possibly due to some twisting in the packing.[1]

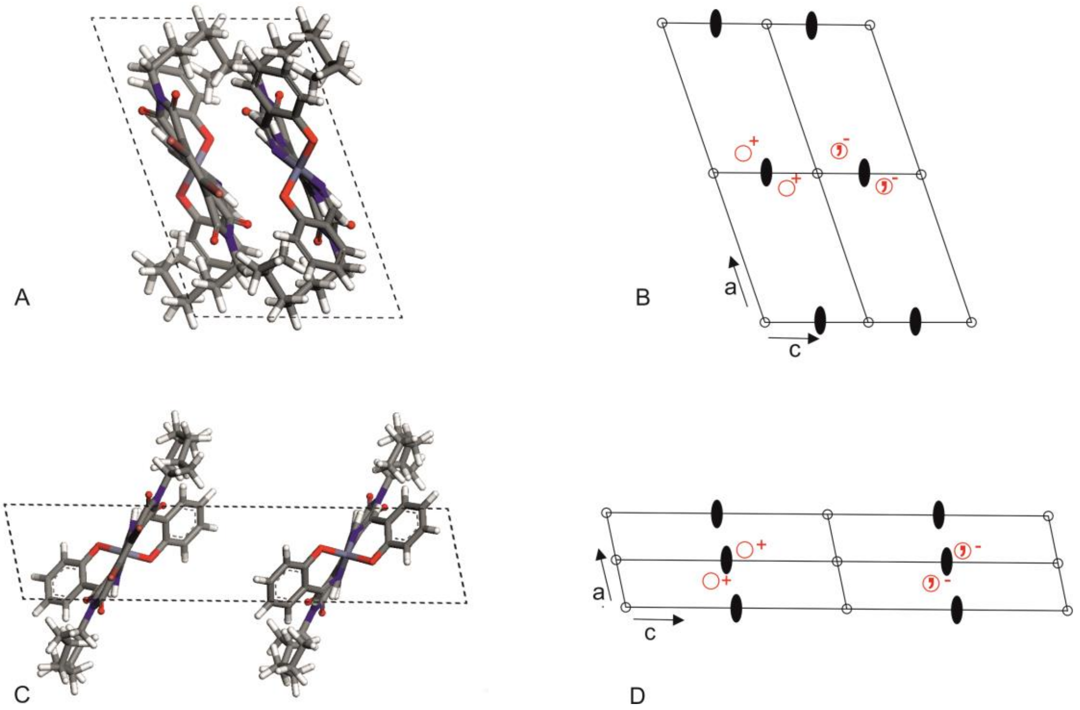

S4 The parallel stacking (A) and antiparallel stacking (C) molecules in the unit cell observed along the 'b' axis with their respective space group diagram (B and D) indicating the symmetry operators for the P2/c space group.

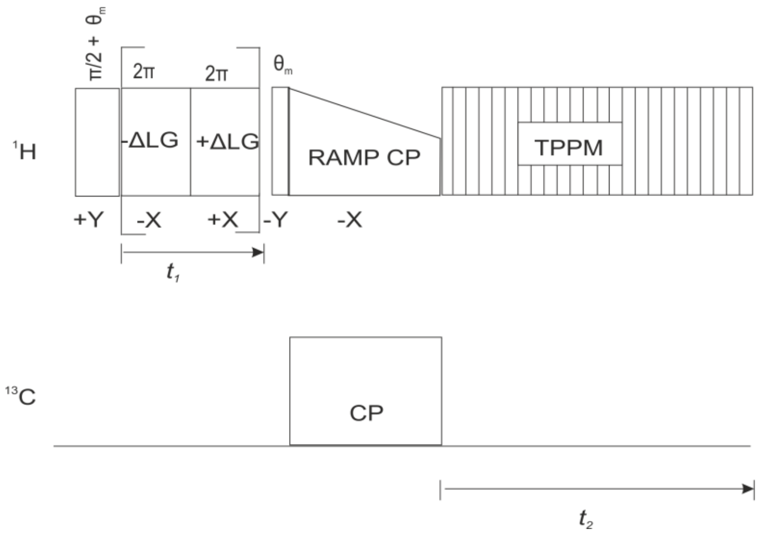

S5 Pulse sequence used for the HETCOR experiment.

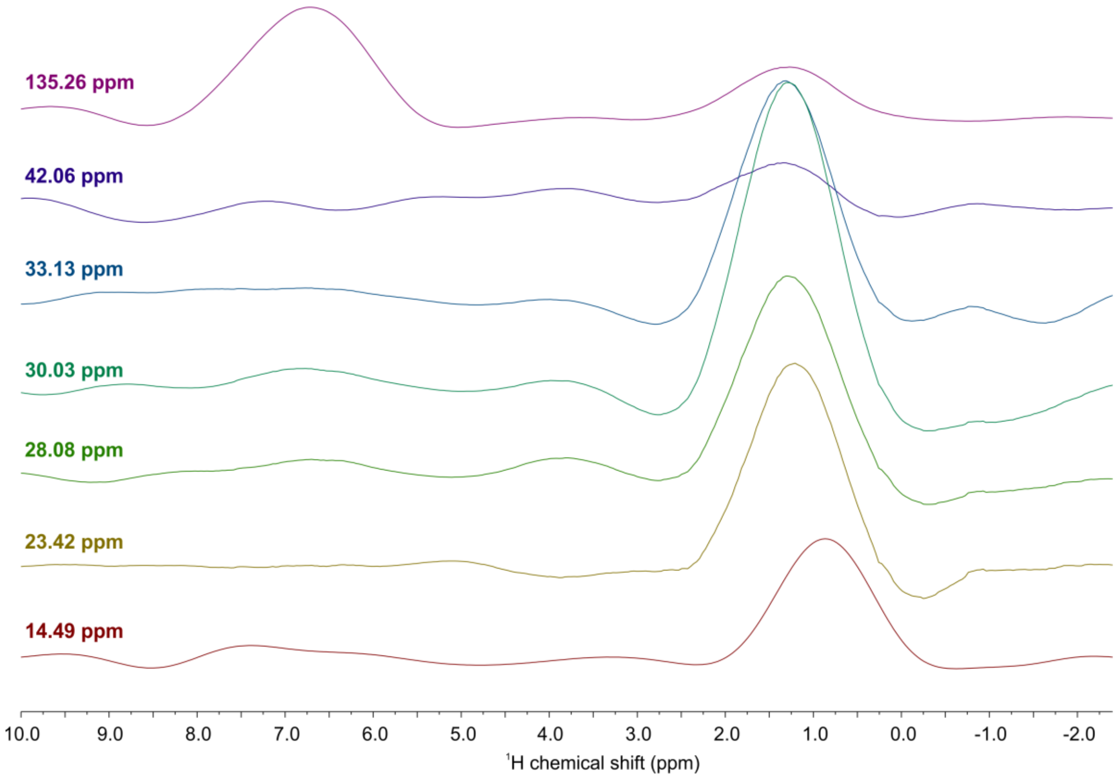

S6 1D slices of peaks at 14 ppm, 23 ppm, 28 ppm, 30 ppm, 33 ppm, 42 ppm and 135 ppm along the ^{1}H dimension.

```
Total enthalpy                    : 182.335351 kcal/mol
  External pressure term          : 0.000000 kcal/mol

Total energy                      :         182.335351 kcal/mol

Contributions to total energy (kcal/mol):
  Valence energy (diag. terms)    :         228.842
    Bond                          :          42.327
    Angle                         :          66.329
    Torsion                       :         115.405
    Inversion                     :           4.782
  Non-bond energy                 :         -46.507
    van der Waals                 :          13.234
    Electrostatic                 :         -59.741

rms force  : 1.277E-004 kcal/mol/A
max force  : 6.247E-004 kcal/mol/A

Cell parameters:    a:  14.752051 A    b:  18.179198 A    c:
9.675065 A
                alpha:  90.000 deg  beta: 109.462 deg gamma:  90.000
deg
```

References

[1] M. R. Hansen, X. Feng, V. Macho, K. Müllen, H. W. Spiess, G. Floudas, *Physical Review Letters* **2011**, *107*, 257801.
